# Pericardial Decompression for Acute Traumatic Cardiac Tamponade—Pearls and Clamshells

**DOI:** 10.1111/1742-6723.70328

**Published:** 2026-08-09

**Authors:** Mark Fitzgerald, Cecil Johnny, Chen Lew, Jeremy Hsu, Christopher Groombridge, Joseph Mathew, Malinda Weerasinghe, Silvana Morasco

**Affiliations:** ^1^ Trauma Service The Alfred Hospital Melbourne Australia; ^2^ National Trauma Research Institute Melbourne Australia; ^3^ Trauma Service Westmead Hospital Sydney Australia; ^4^ Emergency Service Dandenong Hospital Melbourne Australia; ^5^ Cardiothoracic Surgery The Alfred Hospital Melbourne Australia

## Abstract

Acute traumatic cardiac tamponade is a time‐critical—but potentially reversible—cause of circulatory collapse. The surgical, operative, nursing and anaesthesiology staff most capable and best trained to perform the procedure are often not immediately available, requiring emergency medical staff to perform this life‐saving intervention. When tamponade causes refractory shock or peri‐arrest, left anterolateral thoracotomy remains the preferred technique for definitive pericardial decompression, with extension to a clamshell thoracotomy when required.

## 1 Introduction

This article outlines a practical approach for emergency pericardial decompression in patients with cardiac tamponade causing circulatory collapse following thoracic trauma.

## 2 Background

Baron Dominique Larrey described surgical pericardial decompression for cardiac tamponade in his ‘Memoires de Chirurgie’ in 1827 [1]. His technique, developed after cadaveric examination of fatal penetrating thoracic wounds, describes decompressing the pericardium through a left paraxiphoid incision thus avoiding the pleura, with digital identification through the incision prior to incision and drainage.

Larrey had realised that, despite the development of tamponade, not all cardiac wounds continue to bleed. Smaller wounds associated with potential survivors evolve. Haemostasis, hypotension and the pressure within the pericardium secondary to tamponade itself may be initially sufficient to stop bleeding. Relief of the constrictive pressure by drainage of at least some of the accumulated pericardial blood may relieve the tamponade and save the life of the patient.

This concept was further explored over 130 years later at Johns Hopkins in Baltimore in 1949, when a reported series of 8 patients with penetrating thoracic trauma and acute cardiac tamponade were managed with pericardial decompression using needle pericardiocentesis and supportive transfusion alone [2].

With the development of trauma systems during the 1970s, injured patients in extremis began reaching expert medical care earlier. Rapid aeromedical evacuation from scene during the Vietnam war amplified both needle pericardiocentesis and resuscitative thoracotomy as evolving techniques for pericardial decompression secondary to tamponade—and restoration of circulation for patients in circulatory near‐arrest or arrest in the immediate post‐injury phase [3].

The subsequent initial enthusiasm for resuscitative thoracotomy in the Emergency Department, at, or shortly after arrival, had moderated at the time of publication of a 2010–2014 large series by the American College of Surgeons (ACS) Trauma Quality Improvement Program [4]. This study emphasised the infrequent occurrence of acute traumatic tamponade, and reported an overall survival rate of 13.3% for penetrating trauma and 4.4% for blunt injury, with the majority of survivors exhibiting vital signs prior to intervention. An earlier prospective study by the Western Trauma Association had concluded that ED resuscitative thoracotomy was futile if (a) prehospital CPR exceeded 10 min after blunt trauma without a response, (b) prehospital CPR exceeded 15 min after penetrating trauma without a response, and (c) asystole was the presenting rhythm and there was no pericardial tamponade on ultrasound [5].

Emergency Department resuscitative thoracotomy is resource‐intensive—and not without risk to the patient or the attending staff. Also, the surgical, operative, nursing and anaesthesiology staff most capable and best trained to perform the procedure are often not immediately available, requiring emergency medical staff to perform this life‐saving intervention. Institutions with high trauma caseloads have introduced early pericardial decompression as a combined Emergency, Trauma, Cardiothoracic and Anaesthesiology collaboration with survival rates approaching 50% for penetrating trauma and 30% for blunt trauma [6].

## 3 Indications for Emergency Pericardial Decompression

Pericardial decompression is indicated in the adult thoracic trauma patient with thoracic trauma, acute cardiac tamponade and unresponsive circulatory failure. Unresponsive circulatory failure is indicated by persistent hypotension and shock despite oxygenation, pleural decompression, volume restoration and sympathomimetic support [6]. A persistent systolic blood pressure < 70 mmHg despite correction of reversible causes and aggressive resuscitation is a pragmatic threshold prompting urgent decompression. Decompression is likely to be futile if the patient has non‐survivable cranial injuries, no evidence of cardiac activity and/or circulatory arrest of more than 10 min.

## 4 How to Determine if Cardiac Tamponade Is Present

### 4.1 Transthoracic Ultrasound

The widespread availability of point‐of‐care ultrasound established an important diagnostic and triage step for acute traumatic cardiac injury and cardiac tamponade [7]. Ultrasound demonstrated cardiac activity is a key triage tool and indicator for ongoing resuscitative efforts. An underfilled heart with right ventricular collapse is an indicator that the circulating volume is inadequate and filling with blood products is required. Ultrasound may be utilised to then monitor the subsequent response to filling.

Ultrasound may also demonstrate cardiac hypokinesis as a cause for hypoperfusion. This may be secondary to correctible causes including hypothermia, myocardial contusion, traumatic coronary artery injury or coronary ischaemia.

Importantly, ultrasound may demonstrate pericardial fluid in the setting of unresponsive circulatory failure. Pericardial fluid alone does not necessarily indicate the presence of pericardial tamponade. The echocardiographic hallmarks for diagnosing acute cardiac tamponade during trauma resuscitation include pericardial fluid (often with echogenic clot), right atrial collapse during systole and right ventricular collapse during diastole.

There are circumstances when diagnostic images may not be obtained. These include surgical emphysema, disruption of the chest wall anatomy, and user inexperience. Nonetheless, rapid ultrasonographic screening remains an essential first step in the diagnosis and subsequent treatment of cardiac tamponade secondary to trauma.

In a large United Kingdom series (1999–2019) of 601 resuscitative thoracotomies for trauma performed without initial ultrasound screening in the prehospital environment, the overall survival was 30 (5%), with 23 (3.8%) patients surviving with a favourable neurological outcome [8]. 22 of these 23 survivors had cardiac tamponade. Surprisingly, the authors avoided concluding that rapid ultrasound examination would improve decision‐making and reduce unnecessary thoracotomies—despite the in‐hospital realisation of the value of sonographic screening that had already been adopted by 2005.

### 4.2 Digital Exploration

Larrey's initial technique still has conceptual and practical validity. Patients in extremis from penetrating and/or blunt thoracic trauma routinely undergo bilateral pleural decompression as part of their resuscitation. Using the mid‐arm position as a landmark guide, an aseptic, 4–5 cm incision in the left 5th or 6th intercostal space in the anterior axillary line with subsequent double‐gloved, digital pleural decompression may facilitate palpation of the pericardium over the antero‐lateral left ventricle [9].

Digital palpation of the pericardium and left ventricle through this incision will confirm cardiac mechanical activity and—for experienced operators—left ventricular collapse in haemorrhagic shock and pericardial tension in tamponade. However, palpation alone has not been demonstrated as a tool to reliably diagnose tamponade.

## 5 Resuscitative Pericardial Decompression Techniques

As with pleural decompression for tension pneumothorax, the initial goal is to address the immediate life‐threat by identifying pericardial tamponade and performing pericardial decompression. Where available, a key step is contacting surgical, anaesthesiology and operating room teams during the initial reception. Early retrieval activation should occur in centres without immediate access to these resources. Control of the bleeding source—if ongoing bleeding is present—is a mandatory consideration post pericardial decompression.

**FIGURE 1:**
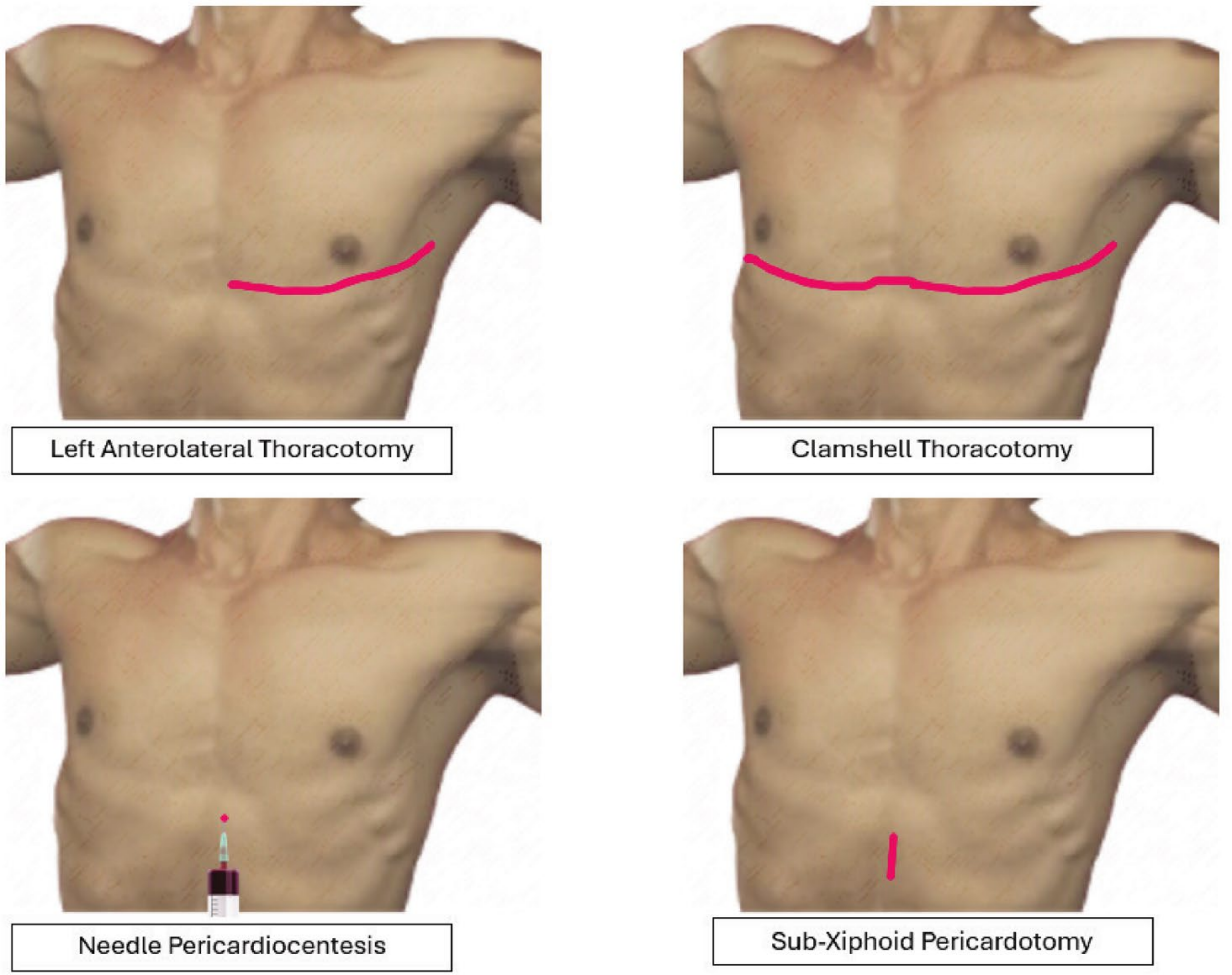
Overview of resuscitative pericardial decompression techniques.

Techniques available for emergency pericardial decompression, each with distinct advantages, limitations and clinical indications, are summarised in Table 1 and Figure 1.

**TABLE 1:** Summary of resuscitative pericardial decompression techniques.

Technique	Advantages	Limitations	Role
Left anterolateral thoracotomy	Provides definitive pericardial decompression and exposure for temporary haemorrhagic control	Requires specific instrumentation and expertise	Preferred initial approach
Clamshell thoracotomy	Provides maximal exposure and access to mediastinal structures	Most invasive	Extension of left anterolateral approach when additional exposure is required
Needle pericardiocentesis	Rapid; minimally invasive; may restore circulation with small‐volume aspiration	Cannot remove clot; risk of cardiac/coronary injury	Temporising measure if thoracotomy is unavailable
Subxiphoid/transdiaphragmatic pericardiotomy	Allows evacuation of fluid and clot without thoracotomy	Limited exposure; technically challenging; does not permit direct cardiac repair	Selected situations; not a substitute for direct thoracotomy

### 5.1 Left Anterolateral Resuscitative Thoracotomy

Left anterolateral resuscitative thoracotomy remains the approach of choice for peri‐arrest trauma patients with cardiac tamponade. It is a technique that requires specific instrumentation, surgical skills and credentialing.

The left anterior pleural decompression incision is described in Section 4.2 Digital exploration (above) can be extended into a left anterolateral thoracotomy. With the left arm abducted, the incision can be extended to the posterior axillary line, allowing retractor insertion and immediate pericardial exposure.

Once the left phrenic nerve is identified over the left lateral pericardium, the pericardium can be incised anteromedial to the left phrenic nerve using a caudal to cephalad longitudinal incision. If tense, the initial incision through the pericardium can be performed with a scalpel. As tamponade may not be visible through the pericardium, the pericardium should be opened to definitively exclude tamponade.

### 5.2 Clamshell Thoracotomy

If additional exposure is required following an initial left anterolateral thoracotomy, the incision can be carried across the sternum to create a clamshell thoracotomy. This approach provides excellent bilateral thoracic exposure when a left anterolateral thoracotomy alone is insufficient.

Clamshell thoracotomy has been advocated as the initial incision of choice in settings where point‐of‐care ultrasound is unavailable and suitable retractors are not immediately accessible. In most contemporary trauma centres, these limitations are uncommon, and left anterolateral thoracotomy remains an appropriate initial approach, with extension to a clamshell thoracotomy when indicated.

### 5.3 Needle Pericardiocentesis

Considered a temporising procedure only until definitive thoracotomy, needle aspiration of as little as 20 cc of pericardial blood has been associated with a return of circulation for patients with circulatory collapse secondary to cardiac tamponade.

Whilst the widespread availability of point‐of‐care ultrasound may have reduced the associated risk of cardiac injury, needle decompression of the pericardium has limitations related to iatrogenic cardiac, coronary artery injury and the inability to remove clot^13^. These risks limit needle pericardiocentesis to selected patients in extremis, and only when immediate surgical decompression is not available.

### 5.4 Subxiphoid Pericardial Decompression

An aseptic subxiphoid incision, with the xiphoid process then reflected superiorly, may also permit pericardial palpation, identification of tamponade and subsequent initial decompression [10]. It is not recommended as a pericardial decompression technique for the pre‐arrest trauma patient during initial reception and resuscitation.

This technique was utilised by Larrey 200 years prior to the introduction of point‐of‐care ultrasound. Kurimoto and colleagues detailed a prospective, blind, subxiphoid pericardiotomy trial that enrolled 83 patients with prehospital arrest or peri‐arrest secondary to cardiac tamponade. The technique was developed to buy time whilst arranging for definitive surgical decompression and repair of the underlying cause.

The subxiphoid approach may be technically challenging and fraught with risk. Similarly, an incision through the central tendon of the diaphragm, utilised intra‐operatively for patients undergoing midline trauma laparotomy, does not allow cardiac exposure to control the source of bleeding, and subsequent sternotomy/thoracotomy will be required.

## 6 The Management of Underlying Injuries

Most survivable cardiac wounds should be initially controlled with digital pressure, whilst correction of rebound hypertension following decompression is addressed by stopping sympathomimetic agents and rapid transfusions. Definitive repair should occur as soon as feasible with cardiothoracic/surgical oversight.

Foley catheter tamponade and/or cardiac stapling are adjunctive, temporising techniques for small lacerations to the ventricles and pulmonary trunk. However, both techniques can extend the injury and should be deferred if digital pressure alone adequately controls bleeding. Atrial tears can be controlled with local pressure and application of a vascular clamp.

## 7 Future Directions and Training Implications

While left anterolateral thoracotomy remains the definitive method of emergency pericardial decompression in appropriately trained trauma centres, the vast majority of emergency departments in Australia and New Zealand do not have immediate access to multidisciplinary teams capable of performing resuscitative thoracotomy.

A tiered systems‐based approach to training should therefore be considered.

All emergency clinicians involved in trauma resuscitation should be proficient in:

- Rapid recognition of obstructive shock due to cardiac tamponade.
- Focused cardiac ultrasound.
- Early activation of trauma retrieval pathways where definitive intervention is unavailable.
- Ultrasound‐guided pericardiocentesis as a temporising intervention when definitive surgical decompression is unavailable.

Emergency departments managing major trauma should additionally develop structured training, credentialling and multidisciplinary simulation programmes for resuscitative thoracotomy, including left anterolateral thoracotomy and extension to clamshell thoracotomy where appropriate.

Further work should evaluate competency standards, simulation‐based training pathways and regional systems of care to improve timely access to definitive pericardial decompression.

## 8 Conclusion

Acute traumatic cardiac tamponade is a time‐critical—but potentially reversible—cause of circulatory collapse. A structured, multidisciplinary approach can restore circulation and improve survival while minimising risk to both patients and staff.

Point‐of‐care ultrasound provides rapid confirmation of cardiac activity and guides diagnosis, while immediate activation of emergency, trauma, anaesthetic and cardiothoracic teams is essential. When tamponade causes refractory shock or peri‐arrest, left anterolateral thoracotomy remains the preferred technique for definitive pericardial decompression, with extension to a clamshell thoracotomy when required.

In centres without immediate thoracotomy capability, prompt recognition of tamponade, early retrieval activation and ultrasound‐guided needle pericardiocentesis may provide an important bridge to definitive care.

## Conflicts of Interest

The authors declare no conflicts of interest.

## Data Availability

Data sharing not applicable to this article as no datasets were generated or analyzed during the current study.
